# Carcinome hépatocellulaire non fibrolamellaire sur foie sain

**DOI:** 10.11604/pamj.2014.18.155.2762

**Published:** 2014-06-18

**Authors:** Salem Bouomrani, Ichrak Kilani, Hanène Nouma, Alaeddine Slama, Maher Beji

**Affiliations:** 1 Service de Médecine Interne, Hôpital Militaire de Gabes 6000, Tunisie; 2 Service de Gastroentérologie, Hôpital Militaire de Gabès 6000, Tunisie

**Keywords:** Carcinome hépatocellulaire, foie sain, cancer, foie non cirrhotique, hepatocellular carcinoma, healthy liver, cancer, non-cirrhotic liver

## Abstract

Le carcinome hépatocellulaire (CHC) survient le plus souvent sur foie de cirrhose. Sa survenue sur un foie sain est exceptionnelle et pose un véritable défit diagnostique pour le clinicien. Nous rapportons l'observation d'un patient de 53 ans, sans antécédents pathologiques notables qui fût admis pour exploration d'une douleur de l'hypochondre droit évoluant depuis quelques mois avec une exacerbation récente, associée à un amaigrissement important et une altération de l’état général. L'examen clinique notait une hépatomégalie ferme et douloureuse. L’échographie abdominale montrait une masse hétérogène du secteur latéral droit du foie faisant 10 cm de grand axe. La TDM abdominale montrait une masse tissulaire, hétérogène, à vascularisation artérielle importante, mesurant 10 cm de diamètre et occupant le secteur latéral droit du foie. Cette tumeur comprime la branche portale droite sans signes d'extension. Il n'y avait pas d'adénopathie ni d’épanchement intra abdominal. La ponction biopsique écho-guidée avait conclu à un CHC non fibrolamellaire. Le bilan biologique, en particulier les transaminases, le taux de prothrombine, l’électrophorèse des protéines sanguine et l'alpha foeto-protéine, était sans anomalies. Les sérologies de l'hépatites virales B et C ainsi que la recherche des auto anticorps spécifiques des hépatites auto immunes et le bilan cuprique étaient aussi négatives. Vue l’âge, le stade avancé de la tumeur et l'altération de l’état général la conduite thérapeutique était de s'abstenir.

## Introduction

Le carcinome hépatocellulaire (CHC) est la tumeur maligne la plus fréquente du foie. Il occupe le sixième rang parmi tous les cancers de l'homme et représente la troisième cause de décès par néoplasie [1]. Il survient dans plus de 80% des cas sur foie de cirrhose [2] ou bien sur d'autres hépatopathies chroniques non cirrhotiques [2, 3]. De rares cas peuvent se développer sur foie antérieurement sain; en particulier le type fibrolamellaire [2]. En dehors de cette forme fibrolamellaire, le CHC sur foie sain reste exceptionnel [4, 5]. Nous en rapportons une observation.

## Patient et observation

**Figure 1:**
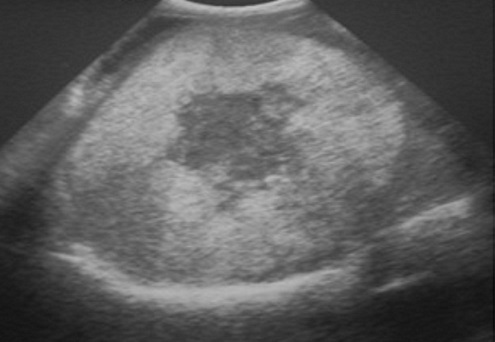
Echographie abdominale: masse hépatique hétérogène du secteur latéral droit

**Figure 2:**
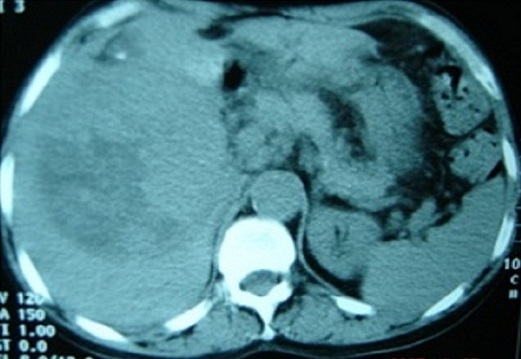
TDM abdominale sans injection de PDC en coupe axiale: masse hépatique hétérogène

**Figure 3:**
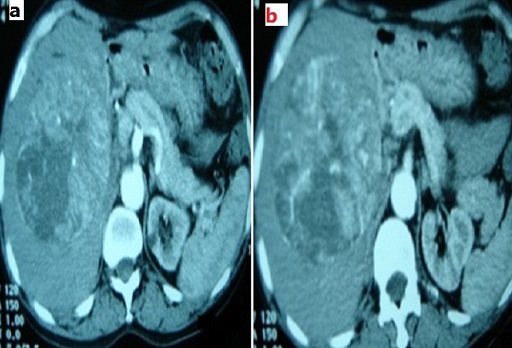
TDM abdominale avec injection de PDC en coupes axiale a et b: aspect hétérogène de la masse hépatique avec une importante vascularisation artérielle

T.I. Patient tunisien âgé de 53 ans, sans antécédents pathologiques notables fût admis pour exploration d'une douleur de l'hypochondre droit évoluant depuis quelques mois avec une exacerbation récente associée à un amaigrissement important et une altération de l’état général. L'examen clinique notait une hépatomégalie ferme et douloureuse. L’échographie abdominale montrait une masse hétérogène du secteur latéral droit faisant 10 cm de grand axe (Figure 1). La TDM abdominale montrait une masse tissulaire, hétérogène, mesurant 10 cm de diamètre, occupant le secteur latéral droit du foie (Figure 2) et qui se caractérise par une vascularisation artérielle importante (Figure 3). Cette tumeur comprime la branche portale droite sans signes d'extension. Il n'y avait pas d'adénopathie ni d’épanchement intra abdominal ni de signes direct ou indirect de cirrhose ou d'hypertension portale. La ponction biopsique écho-guidée avait conclue à un CHC. Il n'a pas été noté de larges hépatocytes éosinophiles ni de bandes fibreuses caractéristiques du carcinome fibrolamellaire. Le bilan biologique, en particulier les transaminases, le taux de prothrombine, le facteur V de l'hémostase, les ‘-glutamyl-transférases, les lactécodéshydrogénases, l’électrophorèse des protéines sanguine, le dosage pondéral des immunoglobulines sériques et l'alpha f'to-protéine, était sans anomalies. Les sérologies des hépatites virales B et C étaient aussi négatives. Le fer sérique, le bilan cuprique (cuprémie, cuprurie et céruloplasmine sérique) et le bilan immunologique (anticorps antinucléaires, anticorps anti mitochondrie type 2, les anticorps anti LKM1 et anticorps anti muscles lisses) étaient dans les limites de la normale éliminant ainsi une hépatopathie chronique sous jacente (hépatite virale chronique B ou C, hémochromatose, maladie de Wilson ou hépatite auto-immune). Vue l’âge, le stade avancé de la tumeur et l'altération de l’état général la conduite thérapeutique était de s'abstenir.

## Discussion

Le CHC sur foie non cirrhotique est rare: 8.8% dans la série de Gomez-Rodriguez R. de 136 cas de CHC [6], 6.2% dans la série de Nunez-Martinez O. de 469 cas de CHC [7] alors qu'il ne représentait que 1.7% dans la grande série de Giannini EG. regroupant 3027 cas de CHC selon les données du registre Italien des cancers du foie [8].

En dehors de la cirrhose quel qu'en soit l’étiologie, le CHC peut survenir au cours d'hépatopathies chroniques non cirrhotiques de plusieurs causes; en particulier l'hépatite virale B chronique [9] même dans ses formes occultes [3], l'hépatite virale C chronique [10, 11], l'hépatite auto-immune [12], le déficit hétérozygote en ‘1 antitrypsine [13], l'hémochromatose hépatique [3, 5] et la stéato-hépatite non alcoolique [14, 15].

Le développement d'un CHC sur foie antérieurement sain (non cirrhotique et en dehors de toute hépatopathie chronique sous jacente) est exceptionnel. Cette éventualité ne représente que 0.32% dans la plus grande série de CHC de la littérature mondiale de 3027 cas de Giannini EG. [8].

Il survient souvent chez le sujet âgé de plus de 60 ans et de sexe masculin [7, 16] mais peut se voir à tout âge; même chez les jeunes de moins de vingt ans [17, 18]. Sa pathogénie n'est pas encore bien élucidée. Plusieurs facteurs de risque pathogéniques sont évoqués tels que les radiations ionisantes, l'exposition à des toxines [5] ainsi que l'adénome hépatique comme lésion précancéreuse potentielle [19].

Une étude moléculaire récente a montré qu'un vieillissement prématuré des cellules souches du foie représente la première étape de la carcinogénèse chez les patients non cirrhotiques. Ce vieillissement prématuré des hépatocytes qui se trouvent fonctionnellement et génétiquement altérées est la conséquence d'une multiplication de dommages génétiques au niveau de l'ADN mitochondrial de ces cellules [20]. De même un dysfonctionnement télomérique secondaire à une accumulation des radicaux libres et des molécules de stress oxydant au cours des hépatopathies chroniques conduit à une surexpression de miR-92, un mico-ARN qui joue un rôle majeur dans la prolifération cellulaire aboutissant à l'immortalisation des hépatocytes et donc à la carcinogénèse hépatique [21, 22].

Un cas anecdotique de CHC sur foie sain chez un jeune militaire de 22 ans a été rapporté [18]. L'activité physique extrême et le stress qui en résulte ainsi que les suppléments nutritionnels protéiques sont suspectés en être responsable [18].

Sur le plan clinique, le CHC sur foie sain n'a pas de symptomatologie spécifique. Il se traduit par des douleurs ou une sensation de pesanteur de l'hypochondre droit mais peut rester asymptomatique dans plus de la moitié des cas [7] expliquant le fait qu'il soit souvent diagnostiqué à un stade avancé et non opérable [8]. L’‘ f'to-protéine peut être élevée ou rester normale [16].

Le diagnostic repose, selon les recommandations mondiales, sur le couple imagerie médicale-histologie. Les aspects radiologiques sont très évocateurs du diagnostic [23]. Les présentations échographiques et tomodensitométriques sont les mêmes que celles du CHC sur foie cirrhotique; la taille supérieure à 2 cm ainsi que la vascularisation artérielle intense sont très suggestives du diagnostic [24].

Le CHC sur foie non cirrhotique se présente classiquement sous forme d'un nodule solitaire: 86.2% des cas dans la série de Nunez-Martinez O. [7] et la taille est plus importante quand il survient sur foie antérieurement sain que quand il se greffe sur foie d'hépatopathie chronique: 7.8 cm en moyenne versus 4 cm [8].

L'imagerie par résonance magnétique (IRM) est jugée supérieure à toutes les autres techniques d'imagerie médicale: échographie, TDM et tomographie par émission de positons (PET-scan) pour le diagnostic des lésions hépatiques focales [25]. L'aspect IRM, en particulier sur les séquences T1, T2, de diffusion et gadolinées avec le gadolénate dimeglumine (Gd-BOPTM) ou l'acide gadoxétique (Gd-EOB) est le plus spécifique du CHC sur foie sain [25].

Le diagnostic différentiel se fait principalement avec les métastases hépatiques, le carcinome fibrolamellaire et l'hyperplasie nodulaire focale du foie (HNF).

L'imagerie permet en plus de faire le bilan d'extension de ces tumeurs qui peuvent métastaser à distance (poumons, ganglions abdominaux, os et c'ur) avec ou sans envahissement de la veine cave inférieure [17].

L'immunohistochimie offre actuellement un outil intéressant et qui parait très prometteur quant à la différentiation du CHC des tumeurs secondaires du foie: c'est l'immunomarquage couplé par l'arginase-1 et l'antigène paraffine-1 hépatocytaire (Hep Par-1) qui se caractérise par une spécificité et une sensibilité marquées [26].

Les options thérapeutiques du CHC sur foie sain sont multiples: chimio-embolisation artérielle, ablation par radiofréquences, tumorectomie et résection hépatiques régularisées [7].

Les hépatectomies régularisées (droite, gauche, droite élargie, segmentaire ou partielle) sont les plus utilisées; particulièrement pour les nodules solitaires [27].

La transplantation hépatique a été aussi tentée [28]. Ses indications dépendent du nombre et de la taille des nodules tumoraux [28] mais ses résultats ne sont pas encourageants [16]. Bien que meilleur que celui du CHC sur foie de cirrhose [16], le pronostic du CHC sur foie sain reste réservé [27] malgré la conservation d'une fonction hépatique sous jacente normale [8]. Les récidives sont fréquentes [16, 27]: leur fréquence est estimée à 57% [29] et la survie à 5 ans n'est que de 50% [16].

L’état général altéré, la consommation de tabac, l'envahissement vasculaire macroscopique, la grande taille de la tumeur ainsi que le traitement non chirurgical sont les facteurs prédictifs d'un mauvais pronostic du CHC sur foie sain [29].

## Conclusion

Le CHC sur foie sain est exceptionnel et de pathogénie encore mal élucidée. Il pose le diagnostic différentiel avec une HNF, une lésion hépatique secondaire ou bien un carcinome fibrolamellaire. Il se caractérise par une grande latence clinique expliquant son diagnostic souvent à un stade avancé et son pronostic réservé. L’échographie de surveillance; surtout chez les sujets avec facteurs de risque, trouve sa place pour le diagnostic précoce de cette tumeur exceptionnelle.
